# The Use of Machine Learning for Analyzing Real-World Data in Disease Prediction and Management: Systematic Review

**DOI:** 10.2196/68898

**Published:** 2025-06-19

**Authors:** Norah Hamad Alhumaidi, Doni Dermawan, Hanin Farhana Kamaruzaman, Nasser Alotaiq

**Affiliations:** 1 College of Medicine Qassim University Buraidah Saudi Arabia; 2 Applied Biotechnology Faculty of Chemistry Warsaw University of Technology Warsaw Poland; 3 Malaysian Health Technology Assessment Section Medical Development Division Ministry of Health Malaysia Wilayah Persekutuan Putrajaya Malaysia; 4 Health Economics and Health Technology Assessment School of Health and Wellbeing University of Glasgow Glasgow United Kingdom; 5 Health Sciences Research Center Imam Mohammad ibn Saud Islamic University Riyadh Saudi Arabia

**Keywords:** machine learning, big data, real-world data, disease prediction, health care management, real-world evidence, artificial intelligence, AI

## Abstract

**Background:**

Machine learning (ML) and big data analytics are rapidly transforming health care, particularly disease prediction, management, and personalized care. With the increasing availability of real-world data (RWD) from diverse sources, such as electronic health records (EHRs), patient registries, and wearable devices, ML techniques present substantial potential to enhance clinical outcomes. Despite this promise, challenges such as data quality, model transparency, generalizability, and integration into clinical practice persist.

**Objective:**

This systematic review aims to examine the use of ML for analyzing RWD in disease prediction and management, identifying the most commonly used ML methods, prevalent disease types, study designs, and the sources of real-world evidence (RWE). It also explores the strengths and limitations of current practices, offering insights for future improvements.

**Methods:**

A comprehensive search was conducted following the PRISMA (Preferred Reporting Items for Systematic Reviews and Meta-Analyses) guidelines to identify studies using ML techniques for analyzing RWD in disease prediction and management. The search focused on extracting data regarding the ML algorithms applied; disease categories studied; types of study designs (eg, clinical trials and cohort studies); and the sources of RWE, including EHRs, patient registries, and wearable devices. Studies published between 2014 and 2024 were included to ensure the analysis of the most recent advances in the field.

**Results:**

This review identified 57 studies that met the inclusion criteria, with a total sample size of >150,000 patients. The most frequently applied ML methods were random forest (n=24, 42%), logistic regression (n=21, 37%), and support vector machines (n=18, 32%). These methods were predominantly used for predictive modeling across disease areas, including cardiovascular diseases (n=19, 33%), cancer (n=9, 16%), and neurological disorders (n=6, 11%). RWE was primarily sourced from EHRs, patient registries, and wearable devices. A substantial portion of studies (n=38, 67%) focused on improving clinical decision-making, patient stratification, and treatment optimization. Among these studies, 14 (25%) focused on decision-making; 12 (21%) on health care outcomes, such as quality of life, recovery rates, and adverse events; and 11 (19%) on survival prediction, particularly in oncology and chronic diseases. For example, random forest models for cardiovascular disease prediction demonstrated an area under the curve of 0.85 (95% CI 0.81-0.89), while support vector machine models for cancer prognosis achieved an accuracy of 83% (*P*=.04). Despite the promising outcomes, many (n=34, 60%) studies faced challenges related to data quality, model interpretability, and ensuring generalizability across diverse patient populations.

**Conclusions:**

This systematic review highlights the significant potential of ML and big data analytics in health care, especially for improving disease prediction and management. However, to fully realize the benefits of these technologies, future research must focus on addressing the challenges of data quality, enhancing model transparency, and ensuring the broader applicability of ML models across diverse populations and clinical settings.

## Introduction

### Background

Advances in big data analytics and the growing availability of real-world data (RWD) are transforming health care by enabling new applications of machine learning (ML) to improve health outcomes [1]. Real-world evidence (RWE) generated from diverse data sources, such as electronic health records (EHRs), patient registries, and wearable devices, has become central to informed decision-making in clinical practice [2,3]. When combined with ML, RWD present a promising avenue to enhance disease prediction, personalize patient management, and optimize therapeutic effectiveness. By providing a comprehensive view of patient histories and real-world health outcomes, ML applications in health care can drive actionable insights across various domains, including disease diagnosis, treatment planning, and chronic disease management [4,5]. RWD capture information about patients in naturalistic settings, revealing how health care is delivered and its outcomes. Unlike clinical trials that operate within controlled conditions, RWD offer a more representative view of patient experiences, treatment responses, and health outcomes [6]. The rise of big data technology and data management systems has facilitated the integration of vast, heterogeneous data types, allowing ML algorithms to identify complex patterns within high-dimensional datasets [7,8]. These capabilities allow health care providers to predict health outcomes, identify at-risk populations, and tailor interventions based on individual patient factors, thus making strides toward precision medicine [9].

Despite their potential, ML applications in RWD and big data contexts face several challenges. Data quality remains a primary concern, as RWD often feature inconsistencies, missing values, and a lack of standardization [10]. Unlike the structured data from controlled clinical trials, RWD demand extensive preprocessing, including advanced natural language processing (NLP) methods and imputation techniques, to address data gaps. Such efforts are critical to enhancing ML model reliability and ensuring accurate, meaningful outcomes [11,12]. Biases present another key issue. ML models trained on RWD may inherit biases from the data, often stemming from demographic imbalances or regional health care differences. If left unaddressed, these biases can lead to health care disparities, as ML-driven decisions might inaccurately represent racial and ethnic minority populations or certain patient groups [13]. Incorporating fairness-aware ML algorithms and cross validating models across multiple datasets can mitigate this challenge, although developing equitable ML models remains a high priority [14]. Another significant hurdle is the interpretability of ML models, especially deep neural network (DNN) models, which are known for their “black box” nature. While complex models deliver high accuracy, their opaque decision-making process limits the ability to verify or explain predictions. Model transparency is crucial given the high stakes in health care, where ML-based recommendations can impact lives. Advances in interpretability tools, such as Shapley Additive Explanations and Local Interpretable Model-Agnostic Explanations, have helped enhance model transparency; however, balancing interpretability with performance remains an area of active investigation [15,16].

The integration of ML with RWD poses ethical and regulatory challenges, especially regarding patient privacy, data security, and informed consent. Regulations such as the Health Insurance Portability and Accountability Act in the United States and the General Data Protection Regulation in the European Union impose strict standards for data protection. However, adapting these laws to the context of ML in health care is complex due to the scale and diversity of the data involved [17,18]. Solutions such as deidentification, secure data-sharing protocols, and clear data management strategies have become crucial to ensuring patient confidentiality while maximizing data utility [19]. Ensuring equitable treatment outcomes is another ethical imperative. ML models trained on data predominantly representing certain demographics may perform poorly on underrepresented groups; therefore, addressing these disparities is critical. By incorporating fairness-aware ML models and building representative datasets, health care practitioners can ensure that ML applications benefit all patient groups, regardless of demographics [20]. Regulatory bodies have started developing specific guidelines for the use of ML and RWD in health care. The Food and Drug Administration (FDA), for example, has issued draft guidance on using RWE for regulatory decisions, and the European Medicines Agency (EMA) has also recognized the importance of RWE in evaluating drug safety and efficacy [21]. As ML applications in health care continue to grow, a solid regulatory framework will be necessary to safeguard patient health while supporting technological innovation.

### Objectives

The objective of this systematic review is to explore and critically analyze the applications, challenges, and future directions of ML in processing real-world health data and big data across various disease domains. Specifically, this review aims to identify the disease areas where ML with RWD has shown clinical utility; examine the ML algorithms and methodologies applied to big data in health care; and analyze the challenges related to data quality, bias, and model interpretability. In addition, this review addresses the ethical and regulatory frameworks pertinent to the use of ML in health care, with an emphasis on patient privacy and fairness. Finally, it outlines future research needs and opportunities for innovation in using ML, RWD, and big data for precision medicine and public health.

## Methods

### Eligibility Criteria

For this systematic review, we focused exclusively on clinical trials and cohort studies that used ML techniques to analyze RWD for disease prediction and management. Studies were included if they met the following criteria: (1) they were randomized controlled trials, pragmatic clinical trials, observational clinical trials, or cohort studies; (2) they involved the application of ML methods (eg, supervised learning, unsupervised learning, and deep learning) to RWD for clinical decision-making, disease prediction, or management of common diseases, such as cardiovascular diseases, diabetes, cancer, and chronic conditions; and (3) they used real-world health data sources, such as EHRs, patient registries, or wearable health devices. Exclusion criteria included trials that did not apply ML techniques or used only data from controlled clinical trials rather than real-world settings.

### Information Sources

The following information sources were used to capture the most relevant clinical trials and cohort studies: PubMed, Scopus, and the Cochrane Library. PubMed was specifically targeted for clinical trials and biomedical research, particularly studies published in leading clinical journals. Scopus and the Cochrane Library were also searched to gather clinical trial reports within the health care and ML domains. To ensure comprehensive coverage, Google Scholar was included to identify gray literature, such as theses and reports that were not indexed in traditional databases. These sources were selected to provide a broad overview of clinical trial data and their relevance to ML applications in disease management. In addition, regulatory bodies such as the US FDA and the EMA were consulted to gain insights into clinical trial guidelines and regulatory standards regarding the use of RWD in health care.

### Search Strategy

A comprehensive search strategy was developed to identify clinical trials and cohort studies focused on ML applications in RWD. The search query incorporated key terms related to ML (eg, “machine learning,” “deep learning,” and “artificial intelligence”) and clinical trials (eg, “clinical trial,” “randomized controlled trial,” and “pragmatic clinical trial”) along with terms related to disease management (eg, “disease prediction,” and “healthcare outcomes”). For example, the search used the following key terms: (“machine learning” OR “deep learning”) AND (“clinical trial” OR “randomized controlled trial” OR “pragmatic trial” OR “cohort study”) AND (“real-world data” OR “electronic health records” OR “patient registries”). Boolean operators (AND and OR), truncation, and Medical Subject Headings terms were used to refine the search and ensure comprehensive coverage. The search was conducted across multiple databases, including the Cochrane Library, PubMed, and Web of Science, covering the period from January 1, 2014, to December 31, 2024, ensuring that the full range of recent literature was captured. In addition, relevant studies were identified through manual searches of reference lists from key articles and by reviewing clinical trial registries, such as ClinicalTrials.gov, to ensure comprehensive coverage of the clinical trials relevant to ML in disease management. Gray literature was identified by conducting targeted searches in Google Scholar and manually retrieving relevant documents suggested by domain experts. Only English-language publications were included. To enhance transparency and reproducibility, the full search strategy, including specific database queries and search filters, has been provided in Multimedia Appendix 1.

### Study Selection

The study selection process was conducted in 2 stages: an initial screening of titles and abstracts by 2 independent reviewers, followed by a full-text review by the same 2 reviewers to ensure consistency and minimize bias. In the first stage, the titles and abstracts of all identified articles were assessed for relevance based on predefined inclusion criteria. Discrepancies between reviewers were resolved through discussion, with a third reviewer consulted when necessary. Studies that met the inclusion criteria proceeded to the second stage, where the full texts were retrieved for further evaluation (Textbox 1).

The evaluation of exclusion criteria was conducted independently by both reviewers, with disagreements resolved through discussion. To maintain transparency, the PRISMA (Preferred Reporting Items for Systematic Reviews and Meta-Analyses) flow diagram [22,23] was used to document the number of studies at each review stage, including identification, screening, eligibility, and final inclusion. The PRISMA checklist is provided in Multimedia Appendix 2.

**Inclusion and exclusion criteria for study selection.**

**Inclusion criteria**

- Study type: clinical trials or cohort studies
- Data source: studies using real-world data, such as electronic health records, patient registries, claims data, or wearable device data
- Machine learning (ML) application: application of ML algorithms for disease prediction or management (eg, supervised, unsupervised, and deep learning models)
- Clinical focus: studies addressing disease prediction, management, monitoring, or outcome prediction in health care
- Outcome reporting: studies reporting ML model performance metrics (eg, accuracy, area under the curve, sensitivity, and specificity) and real-world data sources
- Language: studies published in English
- Publication type: peer-reviewed articles and indexed gray literature

**Exclusion criteria**

- Study type: case reports, cross-sectional studies, reviews, editorials, letters, and conference abstracts
- Data source: studies using simulated data, animal studies, or laboratory-based data
- ML application: studies using only conventional statistical models (eg, logistic regression and Cox models) or expert systems
- Clinical focus: studies unrelated to clinical decision-making, disease outcomes, or patient management
- Outcome reporting: studies lacking sufficient information on ML model performance or data sources
- Language: non–English-language publications
- Publication type: non–peer-reviewed sources (blogs and social media posts)

### Data Extraction

Data extraction was performed independently by 2 reviewers using a standardized form. Key data points extracted from each clinical trial and cohort study included study characteristics (eg, authors, year of publication, and trial design), the specific ML methods used (eg, supervised learning, reinforcement learning, deep learning), disease areas targeted (eg, cardiovascular diseases, diabetes, and cancer), and the types of RWD sources used (eg, EHRs, patient registries, and wearable devices). In addition, we extracted the performance metrics of the ML models used, such as accuracy, sensitivity, specificity, and area under the receiver operating characteristic curve (AUROC), to evaluate their effectiveness in disease prediction and management. Information on the challenges and limitations of applying ML to RWD in clinical trials, such as data quality issues, biases, or model interpretability, was also collected. Any disagreements in data extraction were resolved through discussion. The extracted data were organized systematically to synthesize findings across studies.

## Results

### Systematic Literature Search and Study Selection Workflow

**Figure 1:**
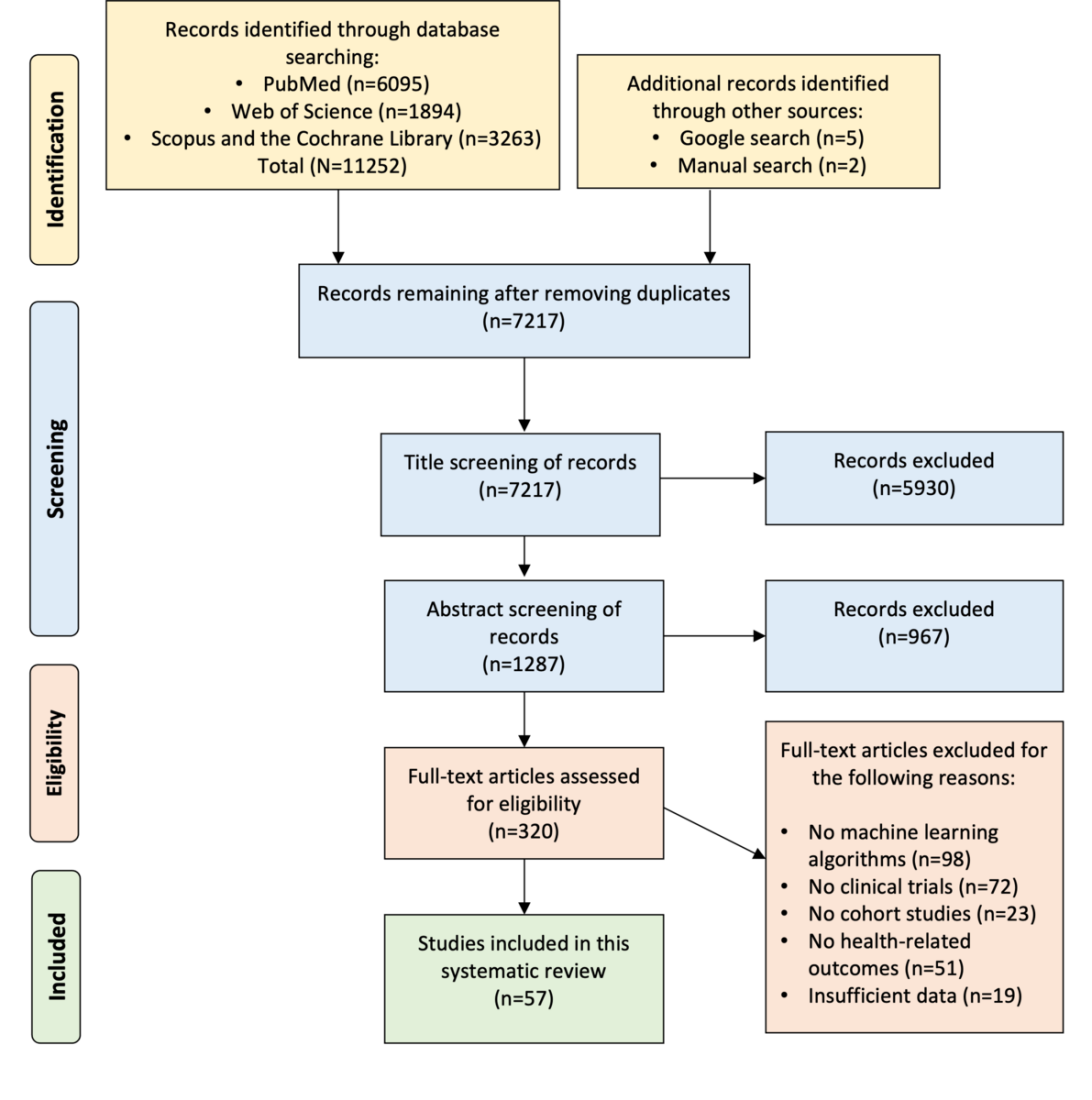
The PRISMA (Preferred Reporting Items for Systematic Reviews and Meta-Analyses) flow diagram depicting the study selection process from initial identification to final inclusion, detailing the number of records screened, excluded, and ultimately included in this systematic review.

The systematic literature search was conducted to identify studies applying ML techniques to RWD in clinical trials and cohort studies, with a focus on disease prediction and management. The search covered multiple databases, including PubMed, Scopus, Web of Science, and the Cochrane Library, to capture a broad range of studies from biomedical, clinical, and health care research fields. This search yielded 11,252 records, as illustrated in the PRISMA flow diagram (Figure 1). To ensure comprehensive coverage, an additional 7 records were identified through external sources such as Google searches, manual hand searching of nonindexed journals, gray literature, and other nontraditional academic sources. After removing duplicates, 7217 (64.13%) unique studies remained for screening. The selection process followed a rigorous, multistage workflow. Title screening was first performed to assess relevance based on predefined inclusion criteria. Studies with titles not indicating the application of ML to RWD in clinical or disease management contexts were excluded, resulting in the removal of 5930 (82.17%) records and leaving 1287 (17.83%) studies for abstract screening. During the abstract screening, each abstract was carefully evaluated for inclusion criteria, including the use of ML techniques, relevant RWD sources (eg, EHRs, patient registries, or wearable device data), and relevance to disease prediction or management. This led to the exclusion of 967 (75.13%) studies that did not meet these criteria, most commonly for the following reasons: (1) a lack of ML implementation, with some studies using only conventional statistical approaches such as logistic regression (LR) or decision trees (DTs) without learning-based model development; (2) irrelevance to clinical trials or cohort study frameworks, instead focusing on simulations, animal studies, or nonhuman data sources; (3) absence of disease prediction or management applications, such as papers limited to health care policy, infrastructure, or economic modeling without patient-centered outcomes; and (4) insufficient use of RWD sources, as studies often used synthetic or trial-generated data rather than EHRs, registries, claims databases, or wearable device data. A total of 320 (24.86%) studies proceeded to the full-text review stage. At this stage, articles were assessed in detail to confirm adherence to all inclusion criteria. A total of 263 studies were excluded for specific reasons: 98 (37.3%) lacked ML algorithms (using conventional statistics instead), 72 (27.4%) were unrelated to clinical trial methodologies, 23 (8.7%) did not involve study cohorts, 51 (19.4%) were unrelated to health care outcomes, and 19 (7.2%) lacked sufficient information on ML model performance or data sources. Following this thorough, systematic, and transparent selection process, 57 studies met all eligibility criteria and were included in the final systematic review. These selected studies represented a diverse range of clinical applications, disease areas, ML methodologies, and RWD sources, offering a comprehensive overview of the current role of ML in clinical trials and cohort studies for disease prediction and management.

Table 1 summarizes these studies, while key findings and methodological details are provided in Multimedia Appendix 3 [24-80].

**Table 1:** A summary of the studies included in this systematic review, outlining the study characteristics, diseases or medical conditions, type of study, source of real-world evidence (RWE), and machine learning (ML) methods used.

Study	Database	Diseases or medical conditions (category)	Type of study	Type of RWE	ML methods	Model performance metrics
Wissel et al [24], 2023	PubMed	Epilepsy (neurological diseases)	Evaluation of health care outcomes	EHR^a^	NLP^b^	AUC^c^=0.79 (95% CI 0.62-0.96) Sensitivity=0.80 (95% CI 0.29-0.99) Specificity=0.77 (95% CI 0.64-0.88) Positive predictive value: 0.25 (95% CI 0.07-0.52) Negative predictive value: 0.98 (95% CI 0.87-1.00).
Ayers et al [25], 2021	PubMed	Orthotopic heart transplantation (CVD^d^)	Survival prediction	Patient registries	DNN^e^, RF^f^, and AdaBoost^g^	RF AUROC^h^=0.691 (95% CI 0.671-0.711) DNN AUROC=0.691 (95% CI 0.671-0.712) AdaBoost AUROC=0.653 (95% CI 0.632-0.674)
Nadarajah et al [26], 2023	PubMed	Atrial fibrillation (CVD)	Disease prediction	EHR	FIND-AF^i^ ML algorithm	AUROC=0.824 (95% CI 0.814-0.834)
Yadgir et al [27], 2022	PubMed	Cognitive impairment (neurological diseases)	Disease prediction	EHR	XGBoost^j^	AUROC=0.720
Liu et al [28], 2023	PubMed	Peripheral artery disease (CVD)	Survival prediction	EHR	LR^k^, GBM^l^, RF, DT^m^, XGBoost, neural network, Cox regression, and RSF^n^	C-index: 0.788 (compared to 0.730 for GermanVasc Score)
Hill et al [29], 2022	PubMed	Atrial fibrillation (CVD)	Disease prediction and cost-effectiveness	EHR	PULSE-AI^o^	Sensitivity=50% Specificity=90%
Sheth et al [30], 2019	PubMed	Acute ischemic stroke (CVD)	Disease prediction	EHR	CNN^p^	AUROC=0.88-0.90
Barton et al [31], 2019	PubMed	Sepsis (infectious diseases)	Disease prediction	EHR	XGBoost	AUROC of 0.88, 0.84, and 0.83 for sepsis onset and 24 and 48 h before onset, respectively
Kao et al [32], 2023	PubMed	Atrial fibrillation (CVD)	Disease prediction	EHR	DT, SVM^q^, LR, and RF	AUROC=0.74 Specificity=98.7%
Kim et al [33], 2022	PubMed	AHREs^r^ (CVD)	Disease prediction	Wearable devices	RF, SVM, LR, and XGBoost	RF AUROC=0.742 SVM AUROC=0.675 XGBoost AUROC=0.745 LR AUROC=0.669
Park et al [34], 2023	PubMed	Coronary artery disease (CVD)	Disease prediction	Patient registries	BQR^s^	AUC of 0.67, 0.65, 0.78, and 0.73 for per‐patient, LAD^t^, LCx^u^, and RCA^v^, respectively
Hilbert et al [35], 2019	PubMed	Acute ischemic stroke (CVD)	Health care outcomes and decision-making	Wearable devices	ResNet^w^	Average AUC for functional outcome was 0.71 Average AUC for reperfusion across all folds was 0.65
Chen et al [36], 2021	PubMed	Ewing sarcoma (tumors)	Survival prediction	Patient registries	Boosted DT, SVM, nonparametric RF, and neural network	Sensitivity=77%-83% Specificity=91%-94%
Koutsouleris et al [37], 2016	PubMed	Schizophrenia (neurological diseases)	Health care outcomes and decision-making	Patient registries	Nonlinear SVM	Test-fold BAC^x^=75%
Strömblad et al [38], 2021	PubMed	Colorectal and gynecologic cancer (cancers)	Health care outcomes	EHR	GBM and LR	—y
Wang et al [39], 2019	PubMed	Atrial fibrillation (CVD)	Decision-making	EHR	DT	—
Tan et al [40], 2021	PubMed	Influenza (infectious diseases and respiratory diseases)	Health care outcomes	EHR	RF, XGBoost, and LR	Accuracy of RF model for hospitalization=0.840, pneumonia=0.765, and sepsis or septic shock=0.857 Accuracy of XGBoost for intensive care unit admission=0.902 Accuracy of LR for in-hospital mortality=0.889
Goerigk et al [41], 2020	PubMed	Depression (neurological diseases)	Decision-making	Patient registries	LR, SVM, RF, tree-based stochastic gradient boosting, and XGBoost	LR: accuracy=0.75, sensitivity=0.76, specificity=0.73, and AUC=0.792 SVM: accuracy=0.88, sensitivity=0.85, specificity=0.91, and AUC=0.939 RF: accuracy=0.89, sensitivity=0.88, specificity=0.91, and AUC=0.957 XGBoost: accuracy=0.88, sensitivity=0.85, specificity=0.91, and AUC=0.954
Kijpaisalratana, et al [42], 2024	PubMed	Sepsis (infectious disease)	Decision-making	EHR	RF, XGBoost, LR, and SVM	AUROC of ML in early sepsis identification was significantly higher than qSOFA^z^, SIRS^aa^, and MEWS^ab^
Sharma et al [43], 2019	PubMed	Acute coronary syndrome (CVD)	Survival prediction	Patient registries	Cox regression	—
Singhal et al [44], 2021	PubMed	ARDS^a^^c^ (respiratory diseases)	Disease prediction	EHR	ML algorithm called “eARDS^ad^” (neural networks, SVM, RF, LR, and XGBoost)	AUROC=0.89 (95% CI 0.88-0.90) Sensitivity=0.77 (95% CI 0.75-0.78) Specificity=0.85 (95% CI 085-0.86)
Kanchanatawan et al [45], 2018	PubMed	Schizophrenia (neurological diseases)	Disease prediction	Patient registries	SVM and RF	SVM AUROC=0.931 RF AUROC=0.898
Huang et al [46], 2022	PubMed	Ischemic stroke (CVD)	Survival prediction	EHR	NB^ae^, XGBoost, and LR	NB AUROC=0.767 XGBoost AUROC=0.989 LR AUROC=0.627
She et al [47], 2023	PubMed	Sepsis (infectious disease)	Disease prediction	Patient registries	SVM and RF	AUC=0.98
Sundar et al [48], 2022	PubMed	Gastric cancer (cancers)	Survival prediction	Patient registries	RF	F-measure: 0.71 AUC=0.75 (95% CI 0.50-0.99)
Alaa et al [49], 2019	PubMed	Cardiovascular disease risk (CVD)	Disease prediction	Patient registries	Linear SVM, RF, neural networks, AdaBoost, and XGBoost	AutoPrognosis model improved risk prediction (AUROC=0.774, 95% CI 0.768-0.780) Framingham score (AUROC=0.724, 95% CI 0.720-0.728; *P*<.001) Cox PH^af^ model with conventional risk factors (AUROC=0.734, 95% CI 0.729-0.739; *P*<.001) Cox PH model with all UK Biobank variables (AUROC=0.758, 95% CI 0.753-0.763; *P*<.001)
Azimi et al [50], 2017	PubMed	LSCS^ag^ (spinal diseases)	Decision-making	EHR	ANN^ah^ and LR	AUC=0.89
Baxter et al [51], 2019	PubMed	Glaucoma (ocular diseases)	Decision-making	EHR	MLR^ai^, RF, and ANN	AUC=0.67
Anderson et al [52], 2015	PubMed	Type 2 diabetes (metabolic diseases)	Disease prediction	EHR	RF and SVM	AUC=0.78
Bannister et al [53], 2018	PubMed	Stroke and myocardial infarction (CVD)	Survival prediction	Patient registries	Cox regression	C-index was 0.59, 0.69, and 0.64 and 0.66, 0.70, and 0.70 for the GP^aj^ and Cox regression models, respectively.
Scheer et al [54], 2017	Web of Science	Spinal deformity surgery (spinal diseases)	Decision-making	Patient registries	DT and ANN	AUROC=0.89
Rau et al [55], 2016	Web of Science	Liver cancer (cancers)	Disease prediction	EHR	ANN and LR	ANN sensitivity=0.757 Specificity=0.755 AUROC=0.873
Ramezankhani et al [56], 2016	Web of Science	Type 2 diabetes (metabolic diseases)	Disease prediction	Patient registries	DT	AUC=0.78 Sensitivity=78%
Pei et al [57], 2019	Web of Science	Type 2 diabetes (metabolic diseases)	Disease prediction	EHR	DT	Accuracy=94.2% Precision=94.0% Recall=94.2% AUC=94.8%
Oviedo et al [58], 2019	Web of Science	Postprandial hypoglycemia (metabolic diseases)	Decision-making	Wearable devices	SVM	Specificity=79% Sensitivity=71%
Mubeen et al [59], 2017	Web of Science	Alzheimer disease (neurological diseases)	Disease prediction	EHR	RF	AUC=0.87 Accuracy=80.2%
Lopez-de-Andres et al [60], 2016	Web of Science	Type 2 diabetes (metabolic diseases)	Survival prediction	Patient registries	ANN	AUROC for Elixhauser comorbidity model=91.7% (95% CI 90.3-93.0) AUROC for Charlson comorbidity model=88.9% (95% CI 87.5-90.2)
Kwon et al [61], 2019	Web of Science	Cardiac arrest (CVD	Survival prediction	Patient registries	DNN, LR, SVM, and RF	DNN AUROC=0.953 (95% CI 0.952-0.954) LR AUROC=0.947 (95% CI 0.943-0.948) RF AUROC=0.943 (95% CI 0.942-0.945) SVM AUROC=0.930 (95% CI 0.929-0.932)
Kim et al [62], 2019	Web of Science	Breast cancer (cancers)	Decision-making	EHR	Two-class decision jungle and 2-class neural network	AUROC was 0.917 in the high RS^ak^ group and 0.744 in the low RS group in the test set
Khanji et al [63], 2019	Web of Science	Hypertension and dyslipidemia (CVD)	Health care outcomes	EHR	LR	cvAUCs^al^ of 0.73 for hypertension, 0.64 for dyslipidemia, and 0.79 for diabetes
Karhade et al [64], 2019	Web of Science	Lumbar disk herniation (spinal diseases)	Decision-making	EHR	LR, RF, XGBoost, ANN, and SVM	AUC=0.81
Jovanovic et al [65], 2014	Web of Science	Choledocholithiasis (gastrointestinal diseases)	Decision-making	EHR	ANN	AUROC=0.884 (95% CI 0.831-0.938); *P*<.001
Kang et al [66], 2020	Web of Science	Postinduction hypotension (anesthesia-related complications)	Health care outcomes	EHR	NB, LR, RF, and ANN	NB AUROC=0.778 (95% CI 0.650-0.898) LR AUROC=0.756 (95% CI 0.630-0.881) RF AUROC=0.842 (95% CI 0.736-0.948) ANN AUROC=0.760 (95% CI 0.640-0.880)
Isma’eel et al [67], 2018	Web of Science	Coronary artery disease (CVD)	Health care outcomes	EHR	ANN	Sensitivity=91% (CI 81%-97%) Specificity=65% (CI 60%-79%)
Hill et al [68], 2019	Web of Science	Atrial fibrillation (CVD)	Disease prediction	EHR	RF, SVM, and Cox regression	RF AUROC=0.827 SVM AUROC=0.725
Dong et al [69], 2019	Web of Science	Chinese Crohn disease (gastrointestinal diseases)	Decision-making	EHR	RF, LR, SVM, DT, and ANN	RF AUROC=0.9864 LR AUROC=0.9538 SVM AUROC=0.9497 DT AUROC=0.8809 ANN AUROC=0.9059
Bowman et al [70], 2018	Web of Science	Carpal tunnel syndrome (musculoskeletal diseases)	Health care outcomes	EHR	LR and ANN	AUROC=0.7
Bertsimas et al [71], 2018	Web of Science	Breast, lung, ovarian cancers (cancers)	Survival prediction	EHR	DT	AUROC=0.83-0.86
Manz et al [72], 2020	The Cochrane Library	Cancer-related serious illness (cancers)	Decision-making	EHR	RF and SVM	—
Tian et al [73], 2023	The Cochrane Library	Lung transplantation (respiratory diseases)	Survival prediction	EHR	RSF	AUROC=0.879 (95% CI 0.832-0.921)
Li et al [74], 2022	The Cochrane Library	Latent profile analysis (cancers)	Decision-making	EHR	GBM	—
Tedeschi et al [75], 2021	The Cochrane Library	Pseudogout (rheumatic diseases)	Disease prediction	EHR	NLP	AUC=0.86
Ambwani et al [76], 2019	The Cochrane Library	Cancer biomarkers (cancers)	Health care outcomes	EHR	LR	Sensitivity=97.3%
Jorge et al [77], 2019	The Cochrane Library	Lupus (autoimmune disease)	Disease prediction	EHR	LR	Specificity=97% Sensitivity=64%
Shimabukuro et al [78], 2017	The Cochrane Library	Sepsis (infectious diseases)	Health care outcomes	EHR	LR	AUROC=0.952 (95% CI 0.946 to –0.958) Specificity=0.900 (95% CI 0.870 to 0.930) Sensitivity=0.900 (95% CI 0.878 to 0.922)
Sarraju et al [79], 2021	The Cochrane Library	Atherosclerosis (CVD)	Health care outcomes	EHR	RF, GBM, XGBoost, and LR	XGBoost AUC of 0.70 (95% CI 0.68 to –0.71) in the full CVD cohort and AUC of 0.71 (95% CI 0.69 to –0.73) in patients with ASCVD^am^, with comparable performance by GBM, RF, and Lasso.
Ye et al [80], 2019	The Cochrane Library	Myopia (ocular diseases)	Health care outcomes	Wearable devices	SVM	AUC=0.99

^a^EHR: electronic health record.

^b^NLP: natural language processing.

^c^AUC: area under the curve.

^d^CVD: cardiovascular disease.

^e^DNN: deep neural network.

^f^RF: random forest.

^g^AdaBoost: adaptive boosting.

^h^AUROC: area under the receiver operating characteristic curve.

^i^FIND-AF: Future Innovations in Novel Detection for Atrial Fibrillation.

^j^XGBoost: Extreme Gradient Boosting.

^k^LR: logistic regression.

^l^GBM: gradient boosting machine.

^m^DT: decision tree.

^n^RSF: random survival forest.

^o^PULSE-AI: Prediction of Undiagnosed Atrial Fibrillation Using a Machine Learning Algorithm.

^p^CNN: convolutional neural network.

^q^SVM: support vector machine.

^r^AHRE: atrial high-rate episode.

^s^BQR: Bayesian quantile regression.

^t^LAD: left anterior descending artery.

^u^LCx: left circumflex artery.

^v^RCA: right coronary artery.

^w^ResNet: residual neural network.

^x^BAC: balanced accuracy.

^y^Not applicable.

^z^qSOFA: quick sequential organ failure assessment.

^aa^SIRS: systemic inflammatory response syndrome.

^ab^MEWS: modified early warning score.

^ac^ARDS: acute respiratory distress syndrome.

^ad^eARDS: early onset acute respiratory distress syndrome

^ae^NB: naive Bayes.

^af^Cox PH: Cox proportional hazards.

^ag^LSCS: lumbar spinal canal stenosis.

^ah^ANN: artificial neural network.

^ai^MLR: multivariable logistic regression.

^aj^GP: genetic programming.

^ak^RS: recurrence score.

^al^cvAUC: cross-validated area under the curve.

^am^ASCVD: atherosclerotic cardiovascular disease.

### Implementation of ML in RWD for Disease Prediction and Management

ML methods have become integral tools for analyzing RWD for disease prediction and management. These methods analyze complex medical data, helping clinicians make informed decisions for better patient care. Random forest (RF) is one of the most widely used ML methods, appearing in 42% (24/57) of the studies (Table 2). It is an ensemble learning technique that builds multiple DTs and combines their outputs to improve model stability and generalizability [81]. Several reviewed studies reported that RF performed well in handling large datasets with numerous variables, particularly EHRs, which are common medical data sources. Its robustness against overfitting and ability to handle missing data made it a frequently chosen method in clinical applications, where data quality could vary [82,83]. While RF has been widely applied in predictive modeling for disease outcomes, treatment responses, and patient risk assessments, further comparative studies are necessary to directly evaluate its performance against other ML models in real-world health care settings [84]. It is often applied to predict disease outcomes, assess treatment responses, and identify patient risk factors.

**Table 2:** The frequency of machine learning (ML) methods used in studies included in this review (N=57).

ML method	Studies, n (%)
Random forest	24 (42)
Logistic regression	21 (37)
Support vector machine	18 (32)
Extreme Gradient Boosting	12 (21)
Artificial neural network	11 (19)
Decision tree	9 (16)
Gradient boosting machine	4 (7)
Cox regression	3 (5)
Natural language processing	2 (4)
Deep neural network	2 (4)

LR was a fundamental method used for binary classification tasks and was used in 37% (21/57) of the studies. LR estimates the probability of a particular class, which is essential for predicting binary outcomes, such as the presence or absence of disease. It is a simple yet powerful tool that works well with smaller datasets and provides results that are easy to interpret, making it particularly useful in clinical settings where transparency is crucial [85,86]. LR is commonly used for disease risk prediction, helping clinicians assess the likelihood of a patient developing a condition based on their medical history and other clinical factors. Its interpretability allows for clear communication of results to health care providers, enhancing decision-making [87,88]. Support vector machine (SVM), used in 32% (18/57) of the studies, is known for its ability to handle high-dimensional data, making it suitable for complex medical datasets, including genomic and imaging data. SVM works by finding the optimal hyperplane that separates different classes in the feature space [89-91]. This method is beneficial in clinical settings where the relationship between variables is nonlinear and can be adapted for classification and regression tasks. SVM is applied in disease prediction, particularly when the dataset has many features relative to the number of observations. It is also useful for classifying patients based on genetic or demographic factors, making it a powerful tool for precision medicine [92,93]. Extreme Gradient Boosting (XGBoost) appeared in 21% (12/57) of the studies and is a highly effective method for improving predictive accuracy through boosting. XGBoost builds models sequentially to correct errors made by previous models and uses regularization to prevent overfitting. This method is effective in handling large datasets, common in clinical studies, and where computational efficiency is essential [94,95]. XGBoost is often used for survival analysis and disease outcome prediction, where it can effectively manage the complexity of large datasets and missing data. Its flexibility allows it to be applied across various disease areas, from cancer prognosis to cardiovascular risk assessment [96,97].

Artificial neural network (ANN) models, used in 19% (11/57) of the studies, are powerful tools for modeling complex, nonlinear relationships in data. With multiple layers of interconnected neurons, an ANN can learn intricate patterns from large datasets. It is widely used in applications that involve unstructured data, such as medical imaging and genetic data, where traditional models might struggle [98,99]. ANN is frequently applied to predict disease progression and response to treatments and identify potential biomarkers. In RWD, an ANN helps identify subtle patterns in complex datasets that simpler models might not capture, such as predicting cancer progression from radiological images [100,101]. DTs, featured in 16% (9/57) of the studies, are straightforward and interpretable models that split data into subsets based on feature values. DT models are highly useful in real-world health care settings, where interpretability is essential for clinical decision-making. They are often applied in clinical decision support systems to guide treatment decisions based on patient data [102,103]. In health care, DTs predict disease outcomes, stratify patients by risk, and recommend treatment plans. Their transparency allows clinicians to understand the decision-making process, which is critical for patient trust and informed consent [104]. Gradient boosting machine (GBM) was used in 7% (4/57) of the studies and is a powerful ensemble method that focuses on correcting errors made by previous models. It is effective in producing highly accurate predictions, particularly in the presence of noisy or incomplete data. GBM is more computationally intensive than other methods, but often outperforms simpler models in accuracy. GBM is particularly useful for predicting disease progression and evaluating treatment efficacy in longitudinal studies, where multiple factors influence outcomes over time [105,106].

NLP, used in 4% (2/57) of the studies, is a subfield of artificial intelligence focused on analyzing unstructured textual data. In health care, NLP extracts relevant information from clinical notes, EHRs, and medical literature. It enables clinicians and researchers to analyze vast amounts of text data to identify trends, predict disease outcomes, and assess treatment effectiveness [107,108]. NLP is crucial in extracting insights from EHRs and other textual data sources. It can help in disease prediction by identifying patterns from patient narratives, diagnostic codes, and clinician notes that would otherwise remain hidden in unstructured formats [109]. Cox Regression, used in 5% (3/57) of the studies, is designed explicitly for survival analysis. It is widely applied in clinical research to model the time of an event, such as the onset of a disease or patient survival. This method is precious for understanding how various predictors affect the risk of an event occurring over time. In RWD, Cox regression is often used in cancer studies and other chronic diseases to predict survival times and assess the impact of different treatment regimens, making it indispensable in clinical trials and outcome-based research [110,111]. DNN models, used in 4% (2/57) of the studies, are a more complex version of ANN with multiple hidden layers. DNN models identify intricate patterns and are increasingly used in health care applications involving large and complex data types, such as medical imaging, genomics, and sensor data. DNN is particularly useful for analyzing high-dimensional data, such as medical images (eg, x-rays and magnetic resonance imaging) or genomic data, where the relationships between variables are complex and nonlinear. It helps identify disease markers and predict outcomes based on these complex datasets [112-114]. The diverse range of ML methods used in RWD for disease prediction and management demonstrates the adaptability of these techniques in clinical practice. From interpretable models such as LR and DTs to more complex methods such as DNN and XGBoost, each ML technique uniquely enhances predictive capabilities. These methods enable health care providers to make more accurate, data-driven decisions, ultimately improving patient outcomes and advancing personalized medicine.

### Distribution of Diseases, Study Types, and RWE Sources in ML Applications

The distribution of disease types in studies using ML for disease prediction and management revealed a strong emphasis on cardiovascular diseases, with 19 (33%) of the 57 studies focusing on various conditions within this category (Table 3).

**Table 3:** Disease categories and the studies included in this review (N=57).

Diseases	Studies, n (%)
Cardiovascular diseases	19 (33)
Cancers and tumors	9 (16)
Neurological diseases	6 (11)
Infectious diseases	5 (9)
Metabolic diseases	5 (9)
Spinal diseases	3 (5)
Gastrointestinal diseases	2 (4)
Ocular diseases	2 (4)
Respiratory diseases	2 (4)
Other diseases	4 (7)

This high representation could be attributed to the multifactorial and complex nature of cardiovascular diseases, which often involve a combination of genetic, environmental, and lifestyle factors. Conditions such as atrial fibrillation, heart transplantation, and peripheral artery disease were prominent in these studies, where advanced ML models were used to enhance predictive accuracy and improve patient management. For instance, studies on heart transplantation and atrial fibrillation highlighted the potential of ML algorithms in survival prediction and early disease detection. A work demonstrated that ensemble models, combining RF, DNN, and adaptive boosting, significantly outperformed traditional LR for predicting 1-year survival rates after orthotopic heart transplantation, with an area under the receiver operating characteristic curve of 0.764 [25]. Meanwhile, another study explored the use of the Future Innovations in Novel Detection for Atrial Fibrillation ML algorithm to identify undiagnosed atrial fibrillation using data from EHRs, aiming to improve early detection and intervention. In addition, studies on peripheral artery disease and atrial fibrillation in older adults underscored the utility of ML models in survival prediction and risk assessment [26]. A study developed a predictive model for amputation-free survival after the revascularization process, with the random survival forest model achieving the highest accuracy in predicting long-term outcomes [28]. Similarly, another study used various ML methods, including DTs and RFs, to predict new-onset atrial fibrillation in older adults, achieving high specificity and performance, particularly with the RF model [32]. Furthermore, the use of ML in acute ischemic stroke, including studies by Sheth et al [30] and Hilbert et al [35], illustrated the growing role of deep learning techniques, such as convolutional neural networks and residual neural networks, in improving diagnostic accuracy and predicting patient outcomes [35]. These advancements in ML could potentially revolutionize clinical decision-making and treatment selection, especially for conditions such as stroke, where rapid and accurate assessment is critical.

A significant (9/57, 16%) portion of studies also targeted cancers and tumors, which were often characterized by their heterogeneity and the need for personalized treatment plans. ML algorithms, such as RF and SVM, enhanced early cancer detection, predicted disease recurrence, and assessed the effectiveness of different treatment protocols, demonstrating great potential in oncology settings. One key area of focus was the prediction of disease outcomes. For instance, a study developed a series of ML models to predict the 5-year survival rate for patients with Ewing sarcoma, a rare type of cancer. Using data from 2332 patients, including various algorithms such as boosted DTs, SVMs, RFs, and neural networks, the study found that the RF method performed best, with impressive sensitivity and specificity. This model is now accessible via a web-based application, providing clinicians a valuable tool for assessing survival probabilities for patients with Ewing sarcoma [36]. Another study used a predictive ML model to improve surgical scheduling in cancer surgeries, specifically for colorectal and gynecologic cancers. The research used gradient boosting and LR techniques to predict surgical durations, reducing operational inefficiencies such as patient wait times and optimizing the use of surgical resources, thereby demonstrating how ML could streamline health care operations while maintaining treatment quality [38]. Furthermore, in survival prediction, a study used an RF model to develop a gene signature that predicted the response of patients with gastric cancer to paclitaxel treatment. Their model, which identified a 19-gene signature, enabled the classification of patients into those who would benefit from the treatment, providing a novel approach to personalized cancer therapy [48].

The studies focusing on neurological diseases, including conditions such as epilepsy, cognitive impairment, and schizophrenia, highlighted the significant impact of ML in improving diagnosis, treatment prediction, and health care outcomes. These studies underscored the potential of ML to personalize patient care and optimize clinical decision-making. For instance, a study investigated the application of NLP embedded in EHR to automate alerts for pediatric patients with epilepsy. This ML-driven clinical decision support system successfully increased referrals for epilepsy surgery, with a marked improvement in presurgical evaluation rates and even higher rates of actual surgery, illustrating how NLP-based interventions could influence health care outcomes by improving referral efficiency and treatment access [24]. Similarly, another study focused on the use of XGBoost, an ML algorithm, to identify older patients in the emergency department at high risk for cognitive impairment. This predictive model, using EHR data, demonstrated high sensitivity and specificity, with the potential to reduce the need for in-person screenings and prioritize patients at high risk. By streamlining screening processes, this approach could enhance the detection of cognitive impairments in older adults, potentially leading to earlier interventions and better management of conditions such as dementia [27]. In schizophrenia, a study developed a nonlinear SVM model to predict treatment outcomes for patients with first-episode psychosis. The model was trained on pretreatment patient-reported data and successfully predicted poor versus good treatment outcomes, thus supporting clinical decision-making in terms of which treatments might be more effective for certain patients and identifying those at risk for nonadherence or poor prognosis [37]. These studies collectively demonstrated how ML methods, such as NLP, XGBoost, and RF, were revolutionizing the management of neurological diseases. By enabling early detection, better prediction of disease outcomes, and more informed decision-making, these tools offered substantial improvements in both clinical and health care settings. Infectious diseases, metabolic diseases, spinal diseases, gastrointestinal diseases, ocular diseases, and respiratory diseases each had a smaller but notable presence in the studies (n≤5). These applications generally focused on disease prediction, early diagnosis, and treatment optimization. ML models such as XGBoost and DNN were used to predict disease onset, assess risks, and improve patient management in these areas.

The data revealed that EHRs were the most frequently used form of RWE, accounting for 68% (39/57) of the studies. EHRs were a rich source of patient data, providing comprehensive records of patient health status, diagnoses, treatments, and outcomes over time. This made EHRs particularly valuable for studies that required large-scale data to identify patterns, trends, and correlations in real-world clinical settings. The next most commonly used type of RWE was patient registries, which were used in 26% (15/57) of the studies. Patient registries typically collect data on specific patient populations with particular diseases or conditions, allowing for longitudinal tracking of disease progression and treatment outcomes. Wearable devices were the least used form of RWE, accounting for 7% (4/57) of the studies. Wearables were increasingly being used to collect real-time health data, including vital signs and activity levels, which could provide valuable insights into patients’ health status outside of clinical environments. This distribution highlighted the dominance of EHR as the primary data source in these studies, reflecting its accessibility and broad applicability in health care research.

Disease prediction emerged as the most widely studied area, represented by 35% (20/57) of the studies. This suggested a strong emphasis on using ML and data analytics to predict the onset, progression, or outcomes of various diseases. The next most studied area was decision-making, with 25% (14/57) of the studies that underscored the growing interest in leveraging data-driven insights to inform clinical decisions and treatment strategies. Health care outcomes, such as quality of life, recovery rates, and adverse events, were the focus of 23% (13/57) of the studies, reflecting the importance of understanding how diseases and treatments affect patients’ overall well-being. Survival prediction, accounting for 19% (11/57) of the studies, was another critical area of research, particularly in oncology and chronic diseases, where predicting patient survival and the effectiveness of interventions could guide clinical decision-making. This distribution indicated that disease prediction and decision-making were central to applying RWE in health care, with a significant focus on improving patient outcomes and guiding treatment strategies.

## Discussion

### Principal Findings

The findings of this study underscore the growing application of ML techniques in RWD for disease prediction and management. The results reveal that ML methods, particularly ensemble models such as RF, play a crucial role in enhancing prediction accuracy and addressing the complexities of large and high-dimensional datasets common in health care. Among the top ML methods used, RF was the most widely used, featured in 42% (24/57) of the studies, showcasing its adaptability to a variety of clinical datasets such as EHRs and patient registries. RF’s ability to handle missing data, its resistance to overfitting, and its effectiveness in managing imbalanced datasets made it a powerful tool in predicting disease outcomes [115,116], such as survival rates and complications in cardiovascular diseases and cancer. Regarding disease types, cardiovascular diseases dominated the studies, with 33% (19/57) of the studies dedicated to predicting outcomes related to heart transplantation, atrial fibrillation, and peripheral artery disease. This concentration is likely attributed to the critical need for predictive tools in the early diagnosis and management of these conditions, which account for a significant burden on health care systems globally [117]. ML applications, such as DNN and random survival forests, have been shown to improve the accuracy of survival predictions, assess treatment responses, and enhance patient stratification. In addition, the study highlights the increasing application of ML in predicting conditions such as cancers, neurological disorders, and infectious diseases. These findings align with the broader trend of using RWD to bridge the gap between clinical trials and actual patient care by making predictions based on RWD sources, such as EHRs and wearable devices. As evidenced in the studies reviewed, ML techniques can process vast amounts of medical data from various sources, facilitating early detection, timely intervention, and improved management of chronic conditions. Furthermore, these advancements in ML applications are subject to increasing regulatory oversight. Agencies such as the US FDA and the EMA are actively exploring frameworks for the approval and regulation of ML-driven tools in health care. These regulations aim to ensure ML models’ safety, efficacy, and transparency, especially in real-world applications where data variability and model interpretability remain key concerns. As regulatory bodies continue to define standards for using RWD and ML in clinical settings, ensuring compliance with FDA and EMA guidelines will be essential for the broader adoption and integration of these technologies into clinical practice.

### Comparison With Prior Work

This systematic review aligns with and extends several recent literature reviews that have explored the application of ML to RWD in health care. Previous studies have highlighted the potential of ML models to transform health care by improving disease prediction and patient management [118,119]. However, our review emphasizes a broader scope by including a wide variety of disease types, from cardiovascular diseases and cancer to neurological and infectious diseases, reflecting the growing versatility of ML tools in clinical settings. A notable comparison can be made with a study that focused on EHRs as the primary data source for ML models, conducted by Miotto et al [120]. While their review identified the challenges associated with EHR-based studies, such as data sparsity and heterogeneity, our study similarly acknowledges these limitations but also expands the discussion to include wearable devices and patient registries as additional data sources. These emerging data sources provide a more complete picture of the patient’s health status, significantly improving model performance and enabling better patient monitoring in real-world settings. Another key comparison is with a review that focused on ML’s role in health care decision-making and its integration into clinical workflows [121]. While the study by Beam and Kohane [121] explored various ML algorithms in health care, our review places a stronger emphasis on the role of ensemble models, such as RF, and their applicability across diverse health care datasets. One of the key contributions of our review, which sets it apart from previous works, is the focus on regulatory challenges associated with the deployment of ML models in clinical practice. While other reviews have discussed technical aspects of ML, we specifically address the urgent need for clearer regulatory frameworks from authorities such as the FDA and EMA to ensure the safe and effective approval of ML models in health care. This is an area that has received limited attention in previous reviews but is critical as health care systems begin to rely more heavily on automated systems for clinical decision-making. Overall, this review builds upon the foundations established by previous literature, offering an updated and comprehensive analysis that incorporates new data sources, encompasses a broader range of diseases, and addresses the challenges of regulatory approval and model interpretability in the context of ML in health care.

### Limitations

This review has several limitations. First, while a comprehensive search strategy was used, it is possible that some relevant studies were missed, particularly those that did not explicitly use the selected keywords or were indexed in databases not included in this search. In addition, this review was limited to English-language publications, which may have excluded relevant studies published in other languages. Another potential limitation is the application of strict inclusion and exclusion criteria, which, while ensuring the relevance and quality of the included studies, may have led to the omission of some studies that could have provided valuable insights. For example, studies with limited methodological details or those focusing primarily on deep learning applications were often excluded due to insufficient validation or performance comparisons. Future research could consider broader inclusion criteria to capture a wider range of studies. Furthermore, the variability in study designs and data sources posed challenges in synthesizing findings across different studies. The included studies used different types of RWD, ranging from EHRs and patient registries to imaging and wearable sensor data. This heterogeneity complicates direct comparisons and generalizability. Finally, while efforts were made to minimize bias through independent study screening by 2 reviewers, inherent biases in study selection and data extraction may still exist. The reliance on published literature also introduces publication bias, as studies with negative or inconclusive results may be underrepresented. Future work could integrate unpublished data sources or conduct meta-analyses to provide a more comprehensive assessment of ML applications in RWD.

### Future Work

While this systematic review aimed for comprehensive coverage by using a broad search strategy across multiple databases and gray literature sources, the large disparity between the number of initially retrieved studies and those meeting the final inclusion criteria highlights an important area for improvement in future research. A more focused, topic-specific keyword strategy, combined with the application of advanced database filters, could increase the precision of future searches by limiting the retrieval of irrelevant studies. In addition, integrating artificial intelligence–assisted search tools and NLP algorithms might further enhance the efficiency of systematic literature reviews by streamlining the identification of eligible studies based on more nuanced criteria. Future reviews may also benefit from targeting more narrowly defined subtopics within the broader field of ML applications in RWD, such as specific disease domains, ML model types, or clinical trial phases. These refinements would likely improve the overall relevance and manageability of retrieved records, ensuring a more efficient screening process and focused synthesis of findings.

### Conclusions

In conclusion, this review highlights the transformative potential of integrating ML techniques with RWD in health care, specifically for disease prediction and management. The use of advanced ML models, such as ensemble methods and deep learning, has demonstrated the ability to enhance predictive accuracy, improve patient stratification, and facilitate more personalized and proactive health care. These advancements are poised to significantly impact clinical decision-making, enabling earlier diagnoses, optimized treatment strategies, and efficient resource allocation. However, despite these promising developments, several challenges remain. Issues related to data quality, generalizability across diverse populations, and the interpretability of complex ML models must be addressed to ensure their effective and widespread application. The lack of transparency in some ML algorithms, which often function as “black boxes,” remains a significant barrier to their integration into clinical workflows. Improving the explainability of these models will be crucial in gaining the trust of health care professionals and enhancing the clinical utility of ML predictions. In addition, regulatory frameworks for ML in health care are still evolving, with clear guidelines needed from regulatory bodies such as the FDA and EMA. This will help ensure that ML models meet safety standards and are deployed in clinical settings in a manner that benefits both health care providers and patients. Furthermore, as health care data becomes increasingly heterogeneous, with sources ranging from EHRs to wearable devices and patient registries, strategies for addressing data inconsistencies and ensuring data quality will be essential. Looking ahead, future research should focus on improving the robustness, transparency, and generalizability of ML models, particularly for underrepresented diseases and diverse patient populations. Establishing ethical and regulatory standards for the use of ML in clinical practice will be crucial for fostering public trust and ensuring equitable access to these innovations. Collaboration among clinicians, data scientists, and policy makers will be key to overcoming these challenges, with the ultimate goal of ensuring that ML-driven advancements in health care lead to improved health outcomes, better care delivery, and more equitable health care systems for all.

## Appendix

Multimedia Appendix 1 Full search strategy.

Multimedia Appendix 2 PRISMA (Preferred Reporting Items for Systematic Reviews and Meta-Analyses) checklist.

Multimedia Appendix 3 A dataset of selected studies, including details such as the year of publication, authors’ names, study title, database, diseases or medical conditions, disease category, study type, real-world evidence type, machine learning methods used, and key findings.

## Abbreviations

ANN: artificial neural network
DNN: deep neural network
DT: decision tree
EHR: electronic health record
EMA: European Medicines Agency
FDA: Food and Drug Administration
GBM: gradient boosting machine
LR: logistic regression
ML: machine learning
NLP: natural language processing
PRISMA: Preferred Reporting Items for Systematic Reviews and Meta-Analyses
RF: random forest
RWD: real-world data
RWE: real-world evidence
SVM: support vector machine
XGBoost: Extreme Gradient Boosting

## Data Availability

All data generated or analyzed during this study are included in this published article and its supplementary information files.
