# Supplementary material for: The Use of Machine Learning for Analyzing Real-World Data in Disease Prediction and Management: Systematic Review
Source: JMIR Med Inform. 2025 Jun 19;13:e68898. doi: 10.2196/68898 (PMC12226786; doi:10.2196/68898)
Supplement: Multimedia Appendix 1 [file medinform_v13i1e68898_app1.docx]

**Multimedia Appendix 1**

**Full Search Strategy**

A comprehensive and systematic search strategy was developed to identify clinical trials and cohort studies focused on machine learning (ML) applications in real-world data (RWD) for disease prediction and healthcare management. The strategy was designed following the Preferred Reporting Items for Systematic Reviews and Meta-Analyses (PRISMA) guidelines to ensure transparency, reproducibility, and methodological rigor.

**Search Terms and Query Construction**

The search incorporated key terms and controlled vocabulary (e.g., Medical Subject Headings [MeSH] terms) to maximize the retrieval of relevant literature. The query focused on three main concepts:

1. Machine Learning Methods
   - Keywords: "machine learning," "deep learning," "artificial intelligence," "supervised learning," "unsupervised learning," "ensemble learning," "reinforcement learning," "neural networks," "support vector machine," "random forest," "gradient boosting," "natural language processing"
   - MeSH Terms: "Machine Learning," "Artificial Intelligence," "Neural Networks (Computer)"
2. Clinical Trial and Cohort Study Designs
   - Keywords: "clinical trial," "randomized controlled trial," "pragmatic clinical trial," "cohort study," "prospective study," "retrospective study"
   - MeSH Terms: "Clinical Trials as Topic," "Randomized Controlled Trials as Topic," "Cohort Studies"
3. Real-World Data Sources and Disease Management
   - Keywords: "real-world data," "electronic health records," "patient registries," "disease prediction," "healthcare outcomes," "biomedical informatics"
   - MeSH Terms: "Electronic Health Records," "Registries," "Outcome Assessment (Health Care)"

The search query used in databases:

( "machine learning" OR "deep learning" OR "artificial intelligence" OR "supervised learning" OR "unsupervised learning" OR "ensemble learning" OR "reinforcement learning" OR "neural networks" OR "support vector machine" OR "random forest" OR "gradient boosting" OR "natural language processing" ) AND ( "clinical trial" OR "randomized controlled trial" OR "pragmatic clinical trial" OR "cohort study" OR "prospective study" OR "retrospective study" ) AND ( "real-world data" OR "electronic health records" OR "patient registries" OR "disease prediction" OR "healthcare outcomes" OR "biomedical informatics" )

Boolean operators (AND, OR), **truncation (e.g., “machine learn*” for broader retrieval)**, and proximity operators were employed to refine and optimize search precision.

| **Database** | **Query** | **Results** |
| --- | --- | --- |
| PubMed | ( "machine learning" OR "deep learning" OR "artificial intelligence" OR "AI" OR "supervised learning" OR "unsupervised learning" OR "ensemble learning" OR "reinforcement learning" OR "neural networks" OR "support vector machine" OR "random forest" OR "gradient boosting" OR "natural language processing" OR "predictive modeling" OR "data mining" ) AND ( "clinical trial" OR "randomized controlled trial" OR "pragmatic clinical trial" OR "cohort study" OR "prospective study" OR "retrospective study" OR "observational study" OR "longitudinal study" OR "clinical research" OR "epidemiological study" ) AND ( "real-world data" OR "electronic health records" OR "patient registries" OR "disease prediction" OR "healthcare outcomes" OR "biomedical informatics" OR "big data" OR "health data" OR "medical records" OR "population health" ) | 6,095 |
| Web of Science | TS=( ("machine learning" OR "deep learning" OR "artificial intelligence" OR "AI" OR "supervised learning" OR "unsupervised learning" OR "ensemble learning" OR "reinforcement learning" OR "neural networks" OR "support vector machine" OR "random forest" OR "gradient boosting" OR "natural language processing" OR "predictive modeling" OR "data mining") AND ("clinical trial" OR "randomized controlled trial" OR "pragmatic clinical trial" OR "cohort study" OR "prospective study" OR "retrospective study" OR "observational study" OR "longitudinal study" OR "clinical research" OR "epidemiological study") AND ("real-world data" OR "electronic health records" OR "patient registries" OR "disease prediction" OR "healthcare outcomes" OR "biomedical informatics" OR "big data" OR "health data" OR "medical records" OR "population health") ) | 1,894 |
| Cochrane Library and SCOPUS | ( ("machine learning":ti,ab,kw OR "deep learning":ti,ab,kw OR "artificial intelligence":ti,ab,kw OR "AI":ti,ab,kw OR "supervised learning":ti,ab,kw OR "unsupervised learning":ti,ab,kw OR "ensemble learning":ti,ab,kw OR "reinforcement learning":ti,ab,kw OR "neural networks":ti,ab,kw OR "support vector machine":ti,ab,kw OR "random forest":ti,ab,kw OR "gradient boosting":ti,ab,kw OR "natural language processing":ti,ab,kw OR "predictive modeling":ti,ab,kw OR "data mining":ti,ab,kw) AND ("clinical trial":ti,ab,kw OR "randomized controlled trial":ti,ab,kw OR "pragmatic clinical trial":ti,ab,kw OR "cohort study":ti,ab,kw OR "prospective study":ti,ab,kw OR "retrospective study":ti,ab,kw OR "observational study":ti,ab,kw OR "longitudinal study":ti,ab,kw OR "clinical research":ti,ab,kw OR "epidemiological study":ti,ab,kw) AND ("real-world data":ti,ab,kw OR "electronic health records":ti,ab,kw OR "patient registries":ti,ab,kw OR "disease prediction":ti,ab,kw OR "healthcare outcomes":ti,ab,kw OR "biomedical informatics":ti,ab,kw OR "big data":ti,ab,kw OR "health data":ti,ab,kw OR "medical records":ti,ab,kw OR "population health":ti,ab,kw) ) | 3,263 |

**Databases and Sources**

To ensure a comprehensive literature review, the search was conducted across multiple high-impact biomedical and clinical databases, including:

- Cochrane Library – For systematic reviews and clinical trial references
- PubMed/MEDLINE – For peer-reviewed biomedical and clinical research
- Web of Science – For multidisciplinary citation tracking and indexing of medical literature

Additionally, manual searches were performed in:

- ClinicalTrials.gov – To identify ongoing and completed clinical trials relevant to ML in RWD
- Reference lists of key articles – To ensure no significant studies were missed

**Inclusion and Exclusion Criteria**

Inclusion Criteria:

- Studies published between January 1, 2014, and December 31, 2024
- Original research articles, clinical trials, and cohort studies evaluating ML in disease management
- Studies that explicitly mention ML models applied to RWD sources (e.g., EHRs, registries, observational datasets)
- Peer-reviewed journal articles and conference papers
- Studies published in English

Exclusion Criteria:

- Review articles, editorials, commentaries, and letters to the editor
- Preclinical, animal, or in vitro studies
- Studies without explicit details on ML implementation
- Studies using synthetic datasets instead of real-world clinical data
- Articles that lack full-text availability

**Search Period and Updates**

The search period was set from January 1, 2014, to December 31, 2024, ensuring that the review captured recent advancements in ML applications in clinical settings.

**Transparency and Reproducibility**

To enhance transparency and ensure reproducibility, the full search strategy, including detailed database-specific queries, search filters, and selection criteria, has been included in a multimedia appendix.
